# Genetic and Serological Analysis of H7N3 Avian Influenza Viruses in Mexico for Pandemic Risk Assessment

**DOI:** 10.3390/v17101376

**Published:** 2025-10-15

**Authors:** Guadalupe Ayora-Talavera, Irma López-Martínez, Gisela Barrera-Badillo, Rodrigo Aparicio-Antonio, Nidia Aréchiga-Ceballos, Anita Aguirre-Barbosa, Rosa Maria Wong-Chew, Daniel Canul-Canul, Mario Solís-Hernández

**Affiliations:** 1 Laboratorio de Virología, Universidad Autónoma de Yucatán, Mérida 97225, Mexico; daniel31canulcanul@gmail.com; 2 Instituto de Diagnóstico y Referencia Epidemiológicos, Mexico City 01480, Mexico; irma.lopez@salud.gob.mx (I.L.-M.); gisela.barrera@salud.gob.mx (G.B.-B.); rodrigo.aparicio@salud.gob.mx (R.A.-A.); nidia.arechiga@salud.gob.mx (N.A.-C.); 3 División de Investigación, Facultad de Medicina, Universidad Nacional Autónoma de México (UNAM), Mexico City 04510, Mexico; 4 Comisión México–Estados Unidos para la Prevención de la Fiebre Aftosa y Otras Enfermedades Exóticas de los Animales (CPA), Servicio Nacional de Sanidad, Inocuidad y Calidad Agroalimentaria (SENASICA), Mexico City 04530, Mexico; mario.solis@senasica.gob.mx

**Keywords:** avian influenza, H7N3, Mexico, risk assessment, hemagglutinin, genetic analysis, serology

## Abstract

Avian influenza A viruses pose ongoing threats to human and animal health, with H7 subtypes causing outbreaks globally. In Mexico, highly pathogenic H7N3 viruses have circulated in poultry since 2012, causing sporadic human infections. Here we analyzed genetic markers in hemagglutinin sequences from Mexican H7N3 isolates and conducted serological assays on human populations with poultry exposure. Our results show conserved avian-like receptor binding sites, thus limiting human adaptation, alongside antigenic drift and acquisition of glycosylation sites likely driven by vaccination. Serological testing of 1103 individuals revealed no detectable antibodies against H7N3, indicating a naïve population. Phylogenetic analyses revealed multiple virus clades circulating regionally. These findings suggest that while current H7N3 viruses have limited capacity for sustained human transmission, the lack of population immunity underscores the importance of continued surveillance and risk assessment to mitigate potential pandemic threats.

## 1. Introduction

Avian influenza A viruses are a constant threat to human and animal health. The pandemic risk at the human–animal interface has been highlighted by several transmission events, some with a high mortality rate between infected individuals, others with sporadic and mild infections [1]. The most recent events are attributed to the avian H5N1 clade 2.3.4.4b, where only in the United States it has caused 70 human infections including a fatal case [2]. Avian influenza A viruses circulate in avian species, including waterfowl and migratory species. For most avian viruses, gene constellation, host adaptability, and species barrier have strongly limited avian-to-human transmission events [3,4]. On the contrary, avian species trade, human exposures by close contact, and worldwide poultry activities are significantly increasing the pandemic risk [5,6].

Worldwide, avian H7 viruses are as broadly distributed as the H5 subtype. Only in recent years, H7NX viruses have caused several outbreaks in countries in Asia (China H7N9: 2013–2015, 2017) and in the Americas (Mexico H7N3: 2012, 2013, 2015–2018; USA, H7N8: 2016; H7N9: 2017) [7,8].

In Mexico, the poultry industry is one of the most important agronomic activities, representing 63% of the national livestock production, where chicken meat production accounts for 34.7% [9]. The increase in domestic chicken consumption is also possible through importation of chickens, where 80% of the total volume originates from the USA [10].

In 2012, Mexico reported for the first time an avian influenza outbreak with a novel H7N3 subtype. The virus overcame the human–host barrier and two cases of human infection with a highly pathogenic avian influenza (HPAI) H7N3 virus were reported from the same outbreak [11]. Even though several eradication and control strategies, including depopulation, vaccination, and enhanced surveillance have been implemented [12], H7N3 circulation is still reported in Mexico [13], but no additional human cases have occurred since 2012.

Reports have documented the high evolutionary rate of these viruses in the avian population and the circulation of several phylogenetic clades with geographic differences [14,15].

An international group of influenza experts created the Tool for Influenza Pandemic Risk Assessment (TIPRA), which is based on the “Influenza Risk Assessment Tool” (IRAT) used by the Centers for Disease Control and Prevention (CDC) [16,17]. TIPRA assesses 10 elements grouped in three broad categories, namely properties of the virus, attributes in the human population, and virus ecology and epidemiology in non-human hosts. As a result of the human cases of HPAI H7N3, Mexico was set to use TIPRA to better handle the H7N3 outbreak. In this study, an intersectoral collaboration led by Mexico’s National Influenza Center (InDRE), and experts across the country, analyzed viral traits of H7N3 viruses isolated since the 2012 outbreak. We describe markers in the hemagglutinin associated with risk for avian-to-human transmission, including receptor binding affinity and host range, human adaptation and increased pathogenicity, and serology data as indicative of population immunity.

## 2. Materials and Methods

### 2.1. Molecular Determinants

The molecular determinants were identified from sequence analysis using all available sequences deposited in the Global Initiative on Sharing All Influenza Data (GISAID) semi-public database. The filter search criteria were influenza A subtype H7N3, species all, country of origin Mexico. The number of sequences available was variable based on the selected gene. All sequences from each gene were aligned using Geneious Prime^®^ 2023.0.4. Incomplete sequences not covering the full coding region were eliminated from the analysis. The search for mutations at key residues for specific genes was performed using Flusurver (https://flusurver.bii.a-star.edu.sg, accessed on 10 May 2024) [18], which identified mutations with phenotypic or epidemiological relevance.

### 2.2. Phylogenetic Analysis

Phylogenetic analysis was performed using sequences available for all genes from North America, Europe, Asia, and Africa (obtained from GISAID). Data were downloaded by gene of interest including metadata (submitter, date of collection, strain, country). Phylogenetic trees were generated with IQTREE Web application, GTR Model, Gamma heterogeneity, invariable sites, and 1000 bootstrap replicates [19].

### 2.3. Serology Studies

Hemagglutination Inhibition (HI) and microneutralization (MN) assays were performed with serum samples collected from people with close contact to poultry, either in farms or backyards. Samples were collected between 2017 and 2025 as part of the active surveillance (targeted surveillance) and passive surveillance (through notification) by the National Service for Agri-Food Health, Safety and Quality (SENASICA). Serum samples were treated for the HI or MN assays according to CDC protocols [20]. Briefly, for HI assays, sera were treated with receptor-destroying enzyme (RDE; Denke-Seiken, Tokio, Japan), and for both assays, sera were heat-inactivated at 56 °C for 30 min before testing.

Viruses used in the assays for serum samples from 2017 and 2018 were A/Mexico/InDRE7218/2012 [11]; for serum samples from 2022 they were A/Mexico/InDRE7218/2012 and A/chicken/Coahuila/CPA-03046-22/2022; for samples from 2023 and 2024 they were A/chicken/Coahuila/CPA-03046-22/2022; and for samples from 2025 they were A/chicken/Coahuila/CPA-03046-22/2022 and A/chicken/3682/Nuevo León/2025.

## 3. Results

**Figure 1:**
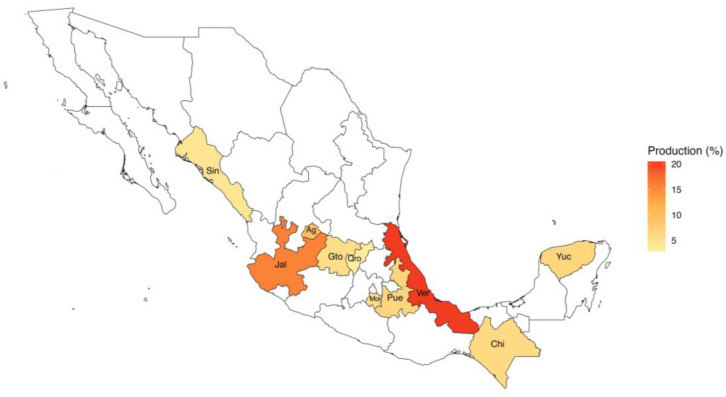
Geographic distribution of chicken production across the top 11 Mexican states, 2021. Sin (Sinaloa); Jal (Jalisco); Ag (Aguascalientes); Gto (Guanajuato); Qro (Querétaro); Mex (Estado de México); Pue (Puebla); Ver (Veracruz); Chis (Chiapas); Yuc (Yucatán). Source: https://una.org.mx/indicadores-economicos/ (accessed on 10 May 2024).

Although the poultry industry in Mexico extends to all the territories, 86% of the national production is concentrated in eleven Mexican states: Veracruz (20.4%), Jalisco (15.5%), Aguascalientes (9.5%), Yucatán (6.43%), Puebla (6.4%), Chiapas (5.7%), Guanajuato (5.14%), Estado de México (5%), Morelos (4.92%), Sinaloa (4.15%), and Querétaro (2.86%) (Figure 1).

The H7N3 virus has been in circulation for twelve years; however, a low number of sequences is available in genetic sequence databases. Overall, a total of 477 sequences from Mexico were downloaded. From all 85 reported isolates, only 57 had full genome sequences, with the highest number of sequences for the HA gene (n = 82), followed by PB1, NP, NA, and M with 57 each, then PB2 (n = 56), and NS (n = 55). Sequences correspond to viruses isolated from chickens in the most affected states: Jalisco (n = 22 sequences), Puebla (n = 11 sequences), Guanajuato (n = 7 sequences), Durango (n = 6 sequences), Coahuila (n = 4 sequences), Aguascalientes (n = 3 sequences), Edo. México (n = 3 sequences), and Querétaro and San Luis Potosí (n = 1 sequence each). The other 21 reported sequences are from Chiapas state isolated from *Ortalis vetula* and *Amazona albifrons*; 2 from Cinnamon Teal ducks (*Spatula cyanoptera*); and 1 sequence corresponding to one of the human cases reported in 2012.

### 3.1. HA Gene and Genetic Markers of Host-Range Restriction and Pathogenicity

The analysis of viral traits in the HA gene was focused on residues with potential effect on the host range, evasion of immune response by glycosylation, and pathogenicity based on multibasic cleavage site.

#### 3.1.1. Characterization of the Receptor Binding Site (RBS)

The RBS on the influenza virus hemagglutinin is composed of three conserved regions: the 130-loop, the 190-helix, and the 220-loop. Changes at the RBS are considered determinants of virus–host adaptation associated with changes in sialic acid preference [21].

The sequence analysis of amino acid residues at these regions in the influenza H7N3 viruses identifies conserved avian-like amino acids with preference to recognizing the α2,3 sialic acid (SA) conformation. The analysis of the 82 HA sequences shows conserved D190, G225, Q226, and G228 (H3 numbering) (Table 1, Figure S1).

To determine how mutations of HA affects its preference for α2-6-linked versus α2-3-linked receptors, a nucleotide analysis was performed to identify by codon usage the potential for any of these amino acid residues to mutate and switch the host restriction of avian H7N3 viruses. At residue 228, 99% of sequences (81 of 82) contain GGA and only 1 sequence of 82 contains GGG. A high prevalence of the nucleotide composition GGA indicates that the virus would require two simultaneous nucleotide changes in the same codon to change from G228S (Table 2). The analysis indicates that RBS changes in this position posed a low risk for the virus to acquire a receptor binding phenotype with preference for the α2-6-linked receptor. These amino acid changes requiring multiple simultaneous mutations are observed in the genomes of naturally occurring H5 isolates, including the recent clade 2.3.4.4b infecting birds and cattle (Table 1). Previous work with H5N1 viruses showed that intermediate sequences failed to bind airway tissues representing mutations that are an evolutionary ‘‘dead end’’ [27].

A second relevant position is residue 226. For H7N3 viruses, the nucleotide composition coding for residue Q226 is CAA, while for H7N9 L226 is CTA. The Q226L change in H7N3 viruses would require a single transversion of the middle nucleotide to change from CAA to CTA to acquire the dual binding capacity observed in H7N9 viruses. However, H7Nx viruses would still require the G228S change for a complete switch to α2,6 SA as observed on human H3N2 viruses.

A third relevant position is residue 186. Although in all H7N3 sequences from Mexico this amino acid is highly conserved, analysis of global H7N3 sequences shows a G186V change in 41 sequences from turkeys isolated around 2004–2006 in Italy and USA, and 32 sequences from ducks isolated around 2018 in USA, Japan, and China.

#### 3.1.2. N-Glycosylation of the HA Protein and Antigenic Drift Mutations

**Figure 2:**
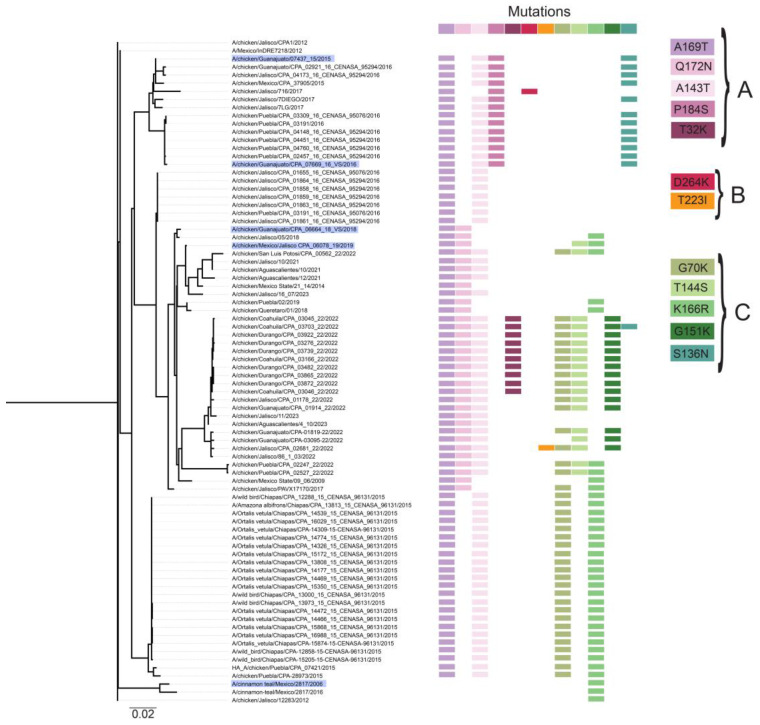
Phylogenetic tree with occurrence of (**A**) glycosylation, (**B**) sites associated with virulence (**C**) antigenic drift changes among H7N3 strains from Mexico, 2012–2023. Vaccine strains are highlighted in blue on the tree leaves.

Glycosylation of the HA significantly affects receptor binding and cleavage of the precursor HA0 protein, influencing the virulence and antigenicity of the virus. The glycosylation on the HA globular head domain physically shields the antigenic sites, preventing antibody recognition and leading to viral evasion from antibody-mediated neutralization [29,30]. In H7N3 viruses, glycosylation may have influenced the evolution of the virus (Figure 2 and Figure S2). After the outbreak in 2012, all viruses isolated in 2014 and later acquired two glycosylation sites, A150T (A169T or A160T/NAA to NAT) and A125T (A135T or A143T/NGA to NGT). Interestingly, these glycosylation sites are also present in viruses isolated in 2015 from wild species in the state of Chiapas. A third glycosylation site Q172N (QMT to NMT) occurred in a cluster of viruses isolated between 2018 and 2023, which coincided with a new vaccine strain (A/chicken/Guanajuato/07437-15/2015) being approved for use in Mexico in 2018. A fourth glycosylation site, P184S, occurred only in viruses circulating during 2016, along with the introduction of vaccine strain A/chicken/Guanajuato/CPA-07669/2016. Notably, we observed the glycosylation loss in site T32K (NGT to NGK) in viruses from Durango and Coahuila collected in 2022, and in site A125T in eight viruses which instead acquired the glycosylation at Q172N (Figure 2). A fifth glycosylation site, T174N, was detected only in four sequences from Jalisco and Guanajuato collected in 2022, instead of Q172N.

A series of mutations related to antigenic drift and escape mutants were also identified (Figure 2). Interestingly, none of these are present on the original viral sequences from 2012. The mutations G70K and T144S only occurred in viruses from 2019 to 2023, forming a cluster. Wild bird viruses also had these changes and grouped with two chicken viruses isolated from Puebla state in 2015 (Figure S1). In viruses from 2022 and 2023, a third mutation, G151K, also associated with antigenic drift and escape mutants, was observed. K166R occurred in the same group of sequences as G70K, except for viruses from 2022 and 2023. This site is highly variable, and the K166R change, associated with the antigenic drift/escape mutant, is present only in 29 sequences; the K166Q change is present in 11 sequences, K166L in 19 sequences, and K166M in 13 sequences (Figures S1 and S3). Finally, mutation S136N (S128N, H3 numbering) was detected only in a cluster of viruses from 2016 isolated from Puebla, Jalisco, and Guanajuato, along with the vaccine strains from 2015 and 2016.

**Figure 3:**
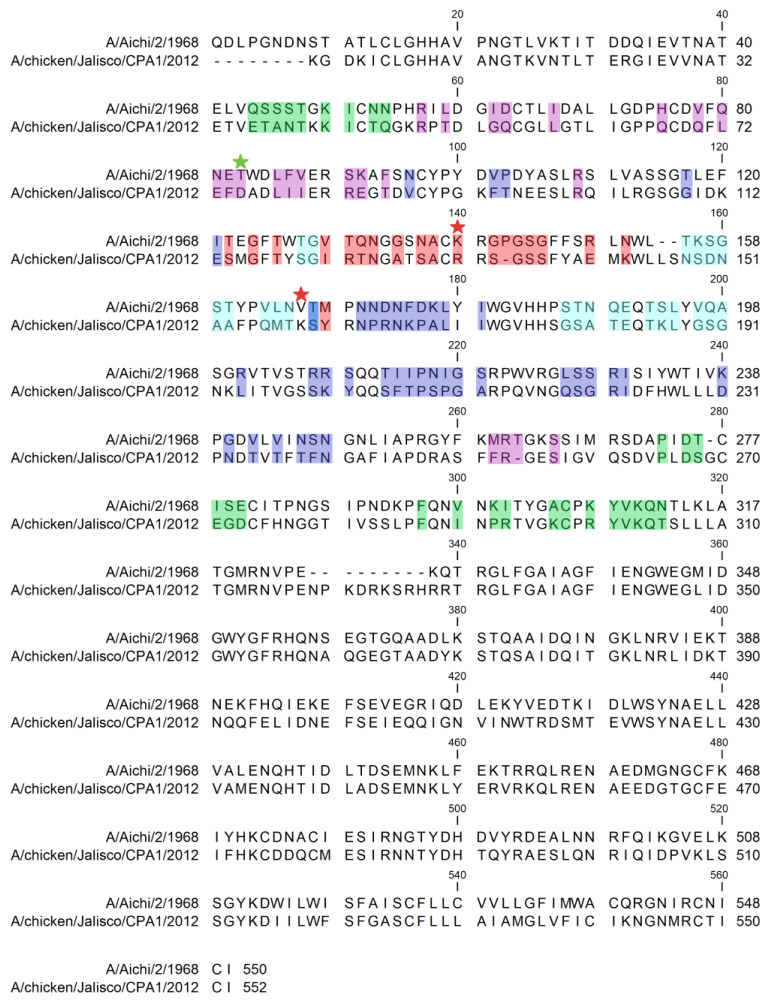
Identification of changes on antigenic sites based on H3 numbering. Red stars indicate positions 140 and 166 located at antigenic sites A (red shade) and B (cyan shade), respectively. The green star indicates position 91 located at antigenic site E (pink shade). Antigenic site C (green shade), antigenic site D (purple shade).

Overall, the frequency of amino acid changes on the HA is observed in those regions related to antigenic sites A and B (Figure 3 and Figure S3). At positions ^140^ RR-SGSS ^146^ (H3 antigenic site A), a high frequency of substitutions associated with escape mutants is observed. Amino acid R140/K/E/T presents high diversity in H7N3 viruses where sequence identity is below 30%. At positions ^156^ SDNAAFPQMTKS ^167^ located in antigenic site Sa in H1 viruses, R166/K/Q/L/M identity is below 30%. A third highly variable position in Mexican H7N3 viruses (below 30% identity) is D91/K/E/G/N, which is located in antigenic site E (H3 numbering).

#### 3.1.3. Multibasic Cleavage Site (MBCS)

**Figure 4:**
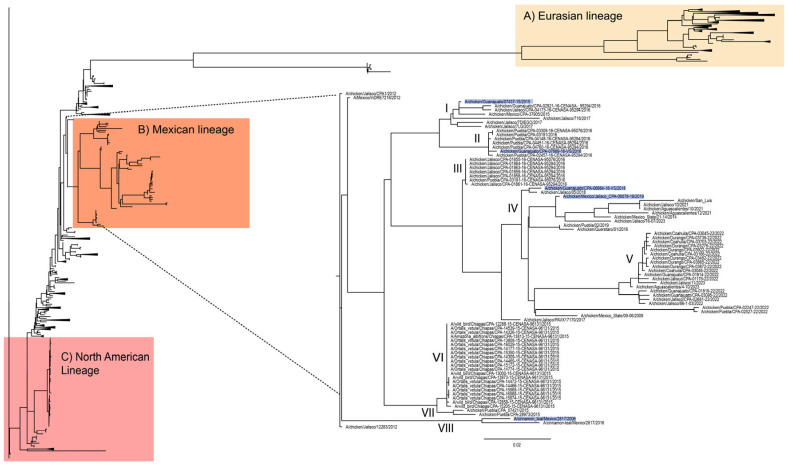
Phylogenetic tree of HA H7N3 viruses worldwide. Mexican H7N3 viruses are grouped by different clades (I–VIII) according to the evolutive divergency. Vaccine strains are blue colored.

Avian H7N3 viruses are characterized by the highly pathogenic (HP) phenotype associated with the insertion of eight basic amino acids at the cleavage site acquired by recombination with the 28S rRNA human host [11,31]. Although some mutations have occurred, the MBCS has remained conserved through time, with changes likely being associated with genetic drift and escape from vaccine strains introduced into poultry populations [14]. Clusters I and II including the vaccine strains from 2015 and 2016 maintain the same multibasic site as viruses from 2012—PENPKDRKSRHRR/GLF—although Puebla viruses showed a change at the fourth amino acid PENPKDRK S/N HRR/GLF, and Jalisco viruses changed at the first two amino acids PENPK D/G R/K/M SRHRR/GLF. Cluster VI is composed of wild bird viruses from 2015 (Figure 4 and Figure S1) and maintained the MBCS as the original viruses. Viruses from 2018–2021, and some from 2022 and 2023 (cluster III and IV), also changed at the first amino acid D/G. The cluster with viruses from the 2022 outbreaks in Durango, Coahuila, Guanajuato, and Jalisco changed at the first and fifth positions PENPK D/S RKS R/Q HRR/GLF, and the cluster with the most recent isolated viruses. Nonetheless, the most recent viruses have not changed their HP phenotype.

#### 3.1.4. Population Immunity Against Avian Influenza H7N3

An important trait to analyze as a risk of human infection with the avian H7N3 virus is population immunity. The results from the analysis of 1103 serum samples showed that none of the people had antibodies against the H7N3 virus. Samples were collected from 23 Mexican states that were reported by active/passive surveillance of suspected cases of avian influenza. The active/passive surveillance was intensified when the H5N1 virus was introduced in Mexico in 2022, and the number of analyzed samples increased up to five times. Samples were collected from different age groups including children, although 78% of all analyzed samples were from adults between 20 and 59 years old, which coincides with most suspected cases originating from either commercial farms or family backyards (Table S1).

### 3.2. Phylogeny of H7N3 Viruses

Overall, there is a high variation in the HA sequences between H7N3 viruses isolated in Mexico from 2016 to 2023. To compare with sequences from other regions, 1161 full HA sequences were downloaded from other regions, including America, Europe, Asia, Africa, and Australia. Phylogenetic analysis of H7N3 viruses including viruses from the most recent outbreaks in 2023 shows significantly divergent branches from the Asian, American, and European viruses (Figure 4 and Figure S4). In fact, eight major clusters are observed within viruses circulating in Mexico. The most recent viruses are reported from Jalisco and Aguascalientes in 2023, grouped in cluster V, along with viruses from 2022 isolated from the states of Durango and Coahuila (Figure 4).

## 4. Discussion

The factors that determine whether an animal influenza virus will cross the host barrier and transmit efficiently are poorly understood; however, the appearance and selection of genetic changes that encode human-adaptive viral traits influence the efficient transmission from avian to humans. Genetic evidence with the HPAI H5N1 virus has underlined that pathogenicity is polygenic. Studying adaptive mutations at the RBS on the virus HA, or mutations/insertions at the HA cleavage site, as well as mutations on the PB2 polymerase, are essential to understanding virus pathogenicity and whether any of these virus attributes can initiate the process of host adaptation.

TIPRA is based on the analysis of ten risk elements to characterize virus attributes and risk for sustained human-to-human transmission [4,32]. This study presents newly generated data for the analysis of factors considered for risk assessment of the HPAI H7N3 viruses circulating in Mexican poultry since their emergence in 2012, which caused two human infections. The data allowed us to describe seven of ten risk elements.

Properties of Viruses. Genetic analysis of the HA indicates that H7N3 viruses have acquired several changes at antigenic sites A and B, but also the incorporation of five glycosylation sites through time, like H7NX viruses circulating in China, suggesting a signature of adaptation among avian reservoirs [33]. However, the genetic composition of H7N3 viruses at the RBS, the predominance of Q226 and G228, and the constraints to mutate suggest a preference for binding to α2-3-linked versus α2-6-linked receptors, which may reduce the risk for human transmission. The genomic analysis for the presence of mutations in NA related to antiviral treatment indicates susceptibility of H7N3 viruses to NA antivirals, although resistance to Amantadine has been reported in viruses from 2012 to 2016 [15] and is still present in recent isolates from Durango and Coahuila from 2023.

The receptor binding properties of influenza viruses are a key determinant of virus–host adaptation. In H1 viruses, including H1N1pdm09, changes at residues E190D and G225D are associated with pathogenicity and avian-to-human adaptation [34,35], whereas changes at Q226L and G228S in avian viruses of the H5 and H7 subtype are associated with receptor binding preference and increased transmission [22,25,36,37]. In human H3N2 viruses, Q226L together with residue 228 have been described as determinants for avian-to-human recognition of SA [22]. In avian H7N9 viruses the natural occurring change Q226L shows a dual profile for 2,3 SA as for 2,6 SA [25], with preference for the former. Seminal work by Dadonaite B et al. (2024) has recently showed through deep mutational scanning that in H5 viruses belonging to clade 2.3.4.4b only two mutations to site Q226/L/R increase HA’s preference for α2-6- versus α2-3-linked sialic acids [28]. Interestingly, in avian H7N9 viruses, G186V together with L226Q showed dual receptor binding for α2,3 SA and α2,6 SA [38,39]. The biological effect of this mutation in the context of avian H7N3 is unknown.

Five glycosylation sites have occurred on H7N3 viruses since the emergency in 2012. These changes suggest an evolutive step to maintain sustained transmission among chickens, but are also likely the result of immune pressure on the virus due to a massive vaccination campaign in 2012 [14]. Glycosylation sites A150T (A169T or A160T/NAA to NAT) are also related to host specificity shift, antigenic drift, and escape mutants in H5 viruses [40].

Several mutations were also detected in H7N3 viruses related to antigenic drift. A mutation in an equivalent position, G70K, has been associated with antigenic drift and escape mutants in avian H5 viruses [41], whereas T144S has been associated with antigenic drift and escape mutants in H3N2 viruses [42] or receptor specificity in H7N7 viruses [43]. Escape mutants with mutation in an equivalent position are reported for human H3 and avian H5 viruses [44,45]. The T144S mutation is reported to be related to antigenic drift and escape in H5N1 viruses [45], and is located on antigenic site Sa in H1 viruses [46]. Mutation S136N is reported to affect antigenicity of human H7N9 viruses when coupled with the A125T glycosylation site [47].

Finally, although some mutations have occurred, the MBCS has remained conserved through the years of virus circulation with changes likely associated with the evolution and diversification into clades after vaccine strains have been introduced into the poultry populations [14].

Attributes in the human population. Two human cases occurred in 2012 with mild infection and ocular symptoms [12]. The analysis of serology indicates no population immunity to the H7N3 virus despite being in circulation for 13 years. Mexico is a country where vaccination has helped control H5N2, H7N3, and more recently, H5N1 outbreaks in poultry farms [12,48,49]. Immune pressure by vaccination may have contributed to genetic drift in the H7N3 virus. Glycosylation has occurred in H7N3 viruses conferring vaccine-induced immune evasion [46], and mutations at key residues on the HA1 segment contributed to maintain viral transmission among the main hosts [50].

Virus ecology and epidemiology in non-human hosts. H7N3 viruses remain a heightened threat for Mexican poultry. The circulation of the H7N3 virus is still confined to avian species, and no additional cases have occurred in humans or other non-human mammals. Some of the mutations, such as A125T occurring in Mexican H7N3 viruses isolated between 2012 and 2016, have not changed the high pathogenicity in chickens, although they have shown that fitness is affected in other avian species [51].

Based on the evidence presented in this study, the traits involved in the adaptation of avian influenza H7N3 viruses at the RBS for efficient human-to-human transmission appear to be limited. Limitations to assess TIPRA were identified, including the lack of studies to investigate the receptor binding properties of recent H7N3 viruses [52] and transmission studies in animal models.

This study presents for the first time data on population immunity against the H7N3 virus. Neutralizing antibodies were not detected, meaning that if H7NX viruses were to acquire the ability to transmit between humans, it would occur in a highly susceptible and naïve human population with potential for extensive spread of infection, besides the two mild previously reported cases [3].

## Figures and Tables

**Table 1:** Comparative nucleotide sequence analysis of key RBS residues in avian viruses associated with switch on sialic acid recognition and risk for human-to-human transmission.

	Amino Acid					SA Receptor	
Virus Subtype	186	190	225	226	228	α2,3	α2,6
H3N2-human [22]		E	G	L	S	−	+++
pH1N1-human [23]		D	D	Q	G	−	+++
H7N7 [24]	G	E	G	Q	G	+++	−
H7N9 [25]	V		G	L	G	+++	+
H7N3 [26]	G	E	G	Q	G	+++	−
H5N1-avian [27]		E	G	Q	G	+++	−
H5N1-2.3.4.4b [28] **		E	G	Q	G	+++	−
				L/R *		++	+

* by pseudovirus deep mutational scanning; ** includes only human viruses; − no binding; +++ strong binding; ++ moderate binding; + low binding assessed by different assays including solid-phase binding assays and/or glycan arrays.

**Table 2:** Nucleotide sequence changes required to switch receptor binding affinity in H7N3 viruses.

	Position 228 ^†^		Position 226
Wt	G(GGA) (99%)		Q(CAA) (100%)
		Wt	
	G(GGG) (1%)		
G228R	R(AGA)	Q226R *	R(CGA)
	R(AGG)		
G228G	G(GGC)		
	G(GGU)		
G228A	A(GCA)		
	A(GCG)		
G228S	S(AGC)		
	S(AGU)	Q226L	L(CTA)
	S(UCA)		
	S(UCG)		

**^†^** Ayora-Talavera et al. [27]; * This mutation was generated by pseudovirus deep mutational scanning showing entry to 293 cells expressing α2-6-linked sialic acids [28]. Wt = wild type.

## Data Availability

Data are provided within the manuscript or Supplementary Information Files. Direct link to sequencing data: (PB2): https://doi.org/10.55876/gis8.250815vq; (PB1): https://doi.org/10.55876/gis8.250815pd; (PA): https://doi.org/10.55876/gis8.250815gk; (HA): https://doi.org/10.55876/gis8.250815hz; (NP): https://doi.org/10.55876/gis8.250815zm; (NA): https://doi.org/10.55876/gis8.250815sa; (MP): https://doi.org/10.55876/gis8.250815cm; (NS): https://doi.org/10.55876/gis8.250815bg.

## Supplementary Materials

The following supporting information can be downloaded at: https://www.mdpi.com/article/10.3390/v17101376/s1, Figure S1: Alignment of hemagglutinin amino acid sequences of Mexican H7N3 viruses; Figure S2: Three-dimensional modeling of avian influenza A(H7N3) hemagglutinin from Mexican isolates; Figure S3: Frequency of amino acids across the hemagglutinin HA1 domain; Figure S4: Phylogenetic trees of all segments; Table S1: Geographic and age distribution of serum samples analyzed by hemagglutination inhibition and microneutralization assays, Mexico, 2017–2019 and 2022–2025.

## Abbreviations

The following abbreviations are used in this manuscript:

HPAI	Highly Pathogenic Avian Influenza
TIPRA	Tool for Influenza Pandemic Risk Assessment
MBCS	Multibasic Cleavage Site
RBS	Receptor Binding Site
HA	Hemagglutinin
NA	Neuraminidase
