# Supplementary material for: Genetic and Serological Analysis of H7N3 Avian Influenza Viruses in Mexico for Pandemic Risk Assessment
Source: Viruses. 2025 Oct 15;17(10):1376. doi: 10.3390/v17101376 (PMC12567671; doi:10.3390/v17101376)
Supplement: Supplementary file 1 [file viruses-17-01376-s001.zip › Data Availability.pdf]

Data Availability

Data is provided within the manuscript or supplementary information files. Direct link to sequencing data:

PB2
GISAID Identifier: EPI_SET_250815vq
DOI: https://doi.org/10.55876/gis8.250815vq
All genome sequences and associated metadata in this dataset are published in GISAID's EpiFlu database. To view the contributors of each individual sequence with details such as accession number, Virus name, Collection date, Originating Lab and Submitting Lab and the list of Authors, visit EPI_SET_250815vq

Data Snapshot
EPI_SET_250815vq is composed of 981 individual viruses.
The collection dates range from 1963-01-01 to 2024-05-20;
Data were collected in 25 countries and territories.

PB1
GISAID Identifier: EPI_SET_250815pd
DOI: https://doi.org/10.55876/gis8.250815pd
All genome sequences and associated metadata in this dataset are published in GISAID's EpiFlu database. To view the contributors of each individual sequence with details such as accession number, Virus name, Collection date, Originating Lab and Submitting Lab and the list of Authors, visit EPI_SET_250815pd

Data Snapshot
EPI_SET_250815pd is composed of 979 individual viruses.
The collection dates range from 1963-01-01 to 2024-05-20;
Data were collected in 24 countries and territories.

PA
GISAID Identifier: EPI_SET_250815gk
DOI: https://doi.org/10.55876/gis8.250815gk
All genome sequences and associated metadata in this dataset are published in GISAID's EpiFlu database. To view the contributors of each individual sequence with details such as accession number, Virus name, Collection date, Originating Lab and Submitting Lab and the list of Authors, visit EPI_SET_250815gk

Data Snapshot
EPI_SET_250815gk is composed of 978 individual viruses.
The collection dates range from 1963-01-01 to 2024-05-20;
Data were collected in 25 countries and territories.

HA
GISAID Identifier: EPI_SET_250815hz
DOI: https://doi.org/10.55876/gis8.250815hz
All genome sequences and associated metadata in this dataset are published in GISAID's EpiFlu database. To view the contributors of each individual sequence with details such as accession number, Virus name, Collection date, Originating Lab and Submitting Lab and the list of Authors, visit EPI_SET_250815hz

Data Snapshot
EPI_SET_250815hz is composed of 991 individual viruses.
The collection dates range from 1963-01-01 to 2024-05-20;
Data were collected in 29 countries and territories.

NP
GISAID Identifier: EPI_SET_250815zm
DOI: https://doi.org/10.55876/gis8.250815zm
All genome sequences and associated metadata in this dataset are published in GISAID's EpiFlu database. To view the contributors of each individual sequence with details such as accession number, Virus name, Collection date, Originating Lab and Submitting Lab and the list of Authors, visit EPI_SET_250815zm

Data Snapshot
EPI_SET_250815zm is composed of 985 individual viruses.
The collection dates range from 1963-01-01 to 2024-05-20;
Data were collected in 25 countries and territories.

NA
GISAID Identifier: EPI_SET_250815sa
DOI: https://doi.org/10.55876/gis8.250815sa
All genome sequences and associated metadata in this dataset are published in GISAID's EpiFlu database. To view the contributors of each individual sequence with details such as accession number, Virus name, Collection date, Originating Lab and Submitting Lab and the list of Authors, visit EPI_SET_250815sa

Data Snapshot
EPI_SET_250815sa is composed of 960 individual viruses.
The collection dates range from 1963-01-01 to 2024-05-20;
Data were collected in 27 countries and territories.

MP
GISAID Identifier: EPI_SET_250815cm
DOI: https://doi.org/10.55876/gis8.250815cm
All genome sequences and associated metadata in this dataset are published in GISAID's EpiFlu database. To view the contributors of each individual sequence with details such as

accession number, Virus name, Collection date, Originating Lab and Submitting Lab and the list of Authors, visit EPI_SET_250815cm

Data Snapshot
EPI_SET_250815cm is composed of 996 individual viruses.
The collection dates range from 1963-01-01 to 2024-05-20;
Data were collected in 25 countries and territories.

NS
GISAID Identifier: EPI_SET_250815bg
DOI: https://doi.org/10.55876/gis8.250815bg
All genome sequences and associated metadata in this dataset are published in GISAID's EpiFlu database. To view the contributors of each individual sequence with details such as accession number, Virus name, Collection date, Originating Lab and Submitting Lab and the list of Authors, visit EPI_SET_250815bg

Data Snapshot
EPI_SET_250815bg is composed of 997 individual viruses.
The collection dates range from 1963-01-01 to 2024-05-20;
Data were collected in 26 countries and territories.
