# Supplementary material for: Genetic and Serological Analysis of H7N3 Avian Influenza Viruses in Mexico for Pandemic Risk Assessment
Source: Viruses. 2025 Oct 15;17(10):1376. doi: 10.3390/v17101376 (PMC12567671; doi:10.3390/v17101376)
Supplement: Supplementary file 1 [file viruses-17-01376-s001.zip › Figure S1.pdf]

```
                                                                                                                                                                                                        130-loop
A/cinnamon_teal/Mexico/2817/2006                    MNTQILVFIACMLIGTKGDKICLGHHAVANGTKVNTLTEKGIEVVNATETVETVNVKKICTQGKRPTDLGQCGLLGTLIGPPQCDQFLEFDADLIIERREGTDVCYPGKFTNEESLRQILRGSGGIDKESMGFTYSGIRTNGATSACRRS
A/chicken/Jalisco/CPA1/2012                         ......AL.......A.......................R.............A.T..............................................................................................
A/Mexico/InDRE7218/2012                             ......AL.......A.......................R.............A.T..............................................................................................
A/chicken/Jalisco/12283/2012                        ......AL.......A.......................R.............A.T..............................................................................................
A/cinnamon-teal/Mexico/2817/2016                    ......A........A................R......................I.........T...................L...................................R............................
A/wild_bird/Chiapas/CPA-12288-15-CENASA-96131/2015  ......AL.......AE......................R.............A.T.....R....K..K..................K..............L.................R...........I........T.......
A/wild_bird/Chiapas/CPA-12858-15-CENASA-96131/2015  ......AL.......AE......................R.............A.T.....R....K..K..................K..............L.................R...........I........T.......
A/wild_bird/Chiapas/CPA-13000-15-CENASA-96131/2015  ......AL.......AE......................R.............A.T.....R....K..K..................K..............L.................R...........I........T.......
/Ortalis_vetula/Chiapas/CPA-13808-15-CENASA-96131/  ......AL.......AE......................R.............A.T.....R....K..K..................K..............L.................R...........I........T.......
A/Amazona_albifrons/Chiapas/CPA-13813-15-CENASA-96  ......AL.......AE......................R.............A.T.....R....K..K..................K..............L.................R...........I........T.......
A/Ortalis_vetula/Chiapas/CPA-14309-15-CENASA-96131  ......AL.......AE......................R.............A.T.....R....K..K..................K..............L.................R...........I........T.......
A/Ortalis_vetula/Chiapas/CPA-14326-15-CENASA-96131  ......AL.......AE......................R.............A.T.....R....K..K..................K..............L.................R...........I........T.......
A/Ortalis_vetula/Chiapas/CPA-14177-15-CENASA-96131  ......AL.......AE......................R.............A.T.....R....K..K..................K..............L.................R...........I........T.......
A/Ortalis_vetula/Chiapas/CPA-14469-15-CENASA-96131  ......AL.......AE......................R.............A.T.....R....K..K..................K..............L.................R...........I........T.......
A/Ortalis_vetula/Chiapas/CPA-14539-15-CENASA-96131  ......AL.......AE......................R.............A.T.....R....K..K..................K..............L.................R...........I........T.......
A/Ortalis_vetula/Chiapas/CPA-14774-15-CENASA-96131  ......AL.......AE......................R.............A.T.....R....K..K..................K..............L.................R...........I........T.......
A/Ortalis_vetula/Chiapas/CPA-15350-15-CENASA-96131  ......AL.......AE......................R.............A.T.....R....K..K..................K..............L.................R...........I........T.......
A/Ortalis_vetula/Chiapas/CPA-15172-15-CENASA-96131  ......AL.......AE......................R.............A.T.....R....K..K..................K..............L.................R...........I........T.......
A/Ortalis_vetula/Chiapas/CPA-16029-15-CENASA-96131  ......AL.......AE......................R.............A.T.....R....K..K..................K..............L.................R...........I........T.......
A/wild_bird/Chiapas/CPA-13973-15-CENASA-96131/2015  ......AL.......AE......................R.............A.T.....R....K..K..................K..............L.................R...........I........T.......
A/Ortalis_vetula/Chiapas/CPA-14466-15-CENASA-96131  ......AL.......AE......................R.............A.T.....R....K..K..................K..............L.................R...........I........T.......
A/Ortalis_vetula/Chiapas/CPA-14472-15-CENASA-96131  ......AL.......AE......................R.............A.T.....R....K..K..................K..............L.................R...........I........T.......
A/Ortalis_vetula/Chiapas/CPA-15868-15-CENASA-96131  ......AL.......AE......................R.............A.T.....R....K..K..................K..............L.................R...........I........T.......
A/Ortalis_vetula/Chiapas/CPA-15874-15-CENASA-96131  ......AL.......AE......................R.............A.T.....R....K..K..................K..............L.................R...........I........T.......
A/Ortalis_vetula/Chiapas/CPA-16988-15-CENASA-96131  ......AL.......AE......................R.............A.T.....R....K..K..................K..............L.................R...........I........T.......
A/wild_bird/Chiapas/CPA-15205-15-CENASA-96131/2015  ......AL.......AE......................R.............A.T.....R....K..K..................K..............L.................R...........I........T.......
A/chicken/Puebla/CPA_07421/2015                     ......AL.....L.AE......................R.............A.T.....R....K..K..................K..............L.................R...........I........T.......
A/chicken/Puebla/CPA-28973/2015                     ......AL.....L.AE......................R.............A.T.....R....K..K..................K..............L.................R.......P...I.N......T.N.....
A/chicken/Jalisco/CPA-01655-16-CENASA-95076/2016    ......AL.....V.A.......................R.............A.T........K.I..E........V......R..K.K........V.....................R....................T....K..
A/chicken/Puebla/CPA-03191-16-CENASA-95076/2016     ......AL.....V.A.......................R.............A.T........K.I..E........V......R..K.K........V.....................R....................T....K..
A/chicken/Jalisco/CPA-01858-16-CENASA-95294/2016    ......AL.....V.A.......................R.............A.T........K.I..E........V......R..K.K........V.....................R....................T....K..
A/chicken/Jalisco/CPA-01859-16-CENASA-95294/2016    ......AL.....V.A.......................R.............A.T........K.I..E........V......R..K.K........V.....................R....................T....K..
A/chicken/Jalisco/CPA-01863-16-CENASA-95294/2016    ......AL.....V.A.......................R.............A.T........K.I..E........V......R..K.K........V.....................R....................T....K..
A/chicken/Jalisco/CPA-01864-16-CENASA-95294/2016    ......AL.....V.A.......................R.............A.T........K.I..E........V......R..K.K........V.....................R....................T....K..
A/chicken/Jalisco/CPA-01861-16-CENASA-95294/2016    ......AL.....V.A.......................R.............A.T........K.I..E........V......R..K.K........V.....................R....................T....K..
A/chicken/Jalisco/PAVX17170/2017                    ......AL..W..V.A.......................R.............A.T.....R....I..E........V......R....K........V...L.......D.........R..................D.T.R..E.L
A/chicken/Guanajuato/CPA-06664-18-VS/2018           ......AL.......A.......................R.............A.I.....RR.K.I..ER.......V......R..K.E........V...L......S..........K..................D.T....E.L
A/chicken/Jalisco/05/2018                           ......AL.......A.......................R.............A.I.....RR.K.I...........V......R....E........V...L.................K..................D.T....E.L
A/chicken/Mexico_State/09-06/2009                   ......AL.......AR......................R.............A.T.....RR.K.I..E........V......R....E........V...L.................K..................D.TS...E.I
A/chicken/Mexico/Jalisco_CPA-06078-19/2019          ......AL.......A.......................R.............A.TN....RR.K.I..E........V......R..K.E........V...L.................K..................D.TSR..E.L
A/chicken/Jalisco/16-07/2023                        ......AL.....T.A.......................R.............A.TN....RR.K.I..K........V......R..K.E........M...L.................K..............E.....TSK..E.L
A/chicken/Puebla/02/2019                            .......L.......A.......................R.............A.T.....RR.K.I..E........V...........K........V...L.................K........I....N....D.TS...E.L
A/chicken/Queretaro/01/2018                         .......L.......A.......................R.............A.T.....RR.K.I..E........V...........K........V...L..............L..K....N..PI....N....D.TS...E.L
A/chicken/San_Luis_Potosi/CPA-00562-22/2022         ......AL...V.T.A.......................R.............A.NN....RNRK.I..K........V......R..K.G........V...L....R............K.........D...R......TSR..E.L
A/chicken/Jalisco/10/2021                           ......AL...V.T.A.......................R.............A.NN....RNRK.I..K........V......R..T.G........V...L.................K.........D...R......TSR..E.L
A/chicken/Aguascalientes/10/2021                    ......AL...V.T.A.......................R.............A.NN....RNRK.I..K........V......R..T.G........V...L.................K.........D...R......TSR..E.L
A/chicken/Aguascalientes/12/2021                    ......AL...V.T.V.......................R.............A.NN....RKRN.I..E...............R..T.G........V...L.................K.............R......TSR..E.L
A/chicken/Mexico_State/21-14/2014                   ......AL.....T.A.......................R.............A.ND....R.RK.I..E........V......R..K.G........V...L.................K.............R....D.TSR..E.L
A/chicken/Puebla/CPA-02247-22/2022                  ......AL.......A.......................R.............A.I.....RRRN.I..K........V......R..A.E........V...L.................K.......P............TSN..K.L
A/chicken/Puebla/CPA-02527-22/2022                  ......AL.......A.......................R.............A.I.....RRRN.I..K........V......R..V.E........V...L.................K.......P............TSN..K.L
A/chicken/Coahuila/CPA-03045-22/2022                ......AL.......A...............K.......R.............A.TR....RR.K.I..K........V.........V.E........V...L.................K....................TSN..K.L
A/chicken/Durango/CPA-03739-22/2022                 ......AL.......A...............K.......R.............A.TR....RR.K.I..K........V.........V.E........V...L.................K....................TSN..K.L
A/chicken/Durango/CPA-03922-22/2022                 ......AL.......A...............K.......R.............A.TR....RR.K.I..K........V.........V.E........V...L.................K....................TSN..K.L
A/chicken/Coahuila/CPA-03166-22/2022                ......AL.......A...............K.......R.............A.TR....RR.K.I..K........V.........V.E........V...L.................K....................TSN..K.L
A/chicken/Coahuila/CPA-03703-22/2022                ......AL.......A...............K.......R.............A.TR....RR.K.I..K........V.........V.E........V...L.................K.............N......TSN..K.L
A/chicken/Coahuila/CPA-03046-22/2022                ......AL.......A...............K.......R.............A.TR....RR.K.I..K........V.........V.E........V...L.................K....................TSN..K.L
A/chicken/Durango/CPA-03482-22/2022                 ......AL.......A...............K.......R.............A.TR....RR.K.I..K........V.........V.E........V...L.................K....................TSN..K.L
A/chicken/Durango/CPA-03865-22/2022                 ......AL.......A...............K.......R.............A.TR....RR.K.I..K........V.........V.E........V...L.................K....................TSN..K.L
A/chicken/Durango/CPA-03872-22/2022                 ......AL.......A...............K.......R.............A.TR....RR.K.I..K........V.........V.E........V...L.................K....................TSN..K.L
A/chicken/Guanajuato/CPA-01914-22/2022              ......AL.......A.......................R.............A.TR....RR.K.I..K........V.........V.E........V...L.................K....................TSN..K.L
A/chicken/Aguascalientes/4-10/2023                  ......AL.......A.......................R.............A.TR....RR.K.I..K........V.........V.E........V...L.................K....................TSN..K.L
A/chicken/Jalisco/11/2023                           ......AL.......A.......................R.............A.TR....RR.K.I..K........V.........V.E........V...L.................K....................TSN..K.L
A/chicken/Durango/CPA-03276-22/2022                 ......AL.......A...............K.......R.............A.TR....RR.K.I..K........V.........V.E........V...L.................K....................TSN..K.L
A/chicken/Jalisco/CPA-01178-22/2022                 ......AL...L...A.......................R.............A.TR....RR.K.I..K........V.........V.E........V...L.................K.......P...N........TSN..K.L
A/chicken/Guanajuato/CPA-01819-22/2022              ......AL.......A.......................R.............A.TR.....R.K.I..K........V...........E........V...L.................K....................TSN..K.L
A/chicken/Jalisco/CPA-02681-22/2022                 ......AL.......A.......................R.............A.TR.....R.K.I..K........V...........E........A...L.................K....................TSN..K.L
A/chicken/Jalisco/86-1-03/2022                      ......AL.......A.......................R.............A.IR.....R.K.I..K........V.S.........E........V...L.................K....................TSN..K.L
A/chicken/Guanajuato/CPA-03095-22/2022              ......AL.......A.......................R.............A.TR...A.R.K.I..N........V...........E........V...L.................K....................TSN..K.L
A/chicken/Guanajuato/07437-15/2015                  ......AL..Y....A.......................R.............A.T..........I..R..................K................................R.........D...N......T....K..
A/chicken/Guanajuato/CPA-02921-16-CENASA-_95294/20  ......AL..Y....A.....................................A.TER........I..R..................K................................R.........D...N......T....K..
A/chicken/Jalisco/CPA-04173-16-CENASA-95294/2016    ......AL..Y....A.......................R.............A.TER........I..R..................K................................R.........D...N......T....K..
A/chicken/Jalisco/7LG/2017                          ......AL..Y....A.......................R.............A.T..........I..R..................K.E..............................R.........D...N......T....I..
A/chicken/Jalisco/7DIEGO/2017                       ......AL..Y....A.......................R.............A.T..........I..R..................K.E........G.....................R.........D...N......T.......
A/chicken/Mexico/CPA-37905/2015                     ......AL..Y....A.......................R.V...........A.I..........I..R..................K................................R.............N......T....K..
A/chicken/Puebla/CPA-03309-16-CENASA-95076/2016     ......AL..Y....A.......................R.............A.I..........I..R...............R..K.N..............................R.........D...N......T....T..
A/chicken/Puebla/CPA-04451-16-CENASA-95294/2016     ......AL..Y....A.......................R.............A.I..........I..R...............R..K.N..............................R.........D...N......T....T..
A/chicken/Puebla/CPA-04760-16-CENASA-95294/2016     ......AL..Y....A.......................R.............A.I..........I..R...............R..K.N..............................R.........D...N......T....T..
A/chicken/Puebla/CPA-02457-16-CENASA-95294/2016     ......AL..Y....A.......................R.............A.I..........I..R...............R..K.N..............................R.........D...N......T....T..
A/chicken/Puebla/CPA-03191/2016                     ......AL..Y....A.......................R.............A.I..........I..R...............R..K.N..............................R.........D...N......T....T..
A/chicken/Puebla/CPA-04148-16-CENASA-95294/2016     ......AL..Y....A.......................R.............A.I..........I..R...............R..K.N..............................R.........D...N......T....T..
A/chicken/Guanajuato/CPA-07669-16-VS/2016           ......AL..Y....A.......................R.............A.I..........I..R...............R..K.N..............................R.........D...N......T....T..
A/chicken/Jalisco/716/2017                          ......AL..Y............................R.............A.T..........I.........................................R............R.............D....D.T....E.F
```

```
                                                                                                190-helix                             220-loop
A/cinnamon_teal/Mexico/2817/2006                      GSSFYAEMKWLLSNSDNAAFPQMTKSYRNPRNKPALIIWGVHHSGSAAEQTKLYGSGNKLITVGSSKYQQSFTPSPGARPQVNGQSGRIDFHWLLLDPNDTVTFTFNGAFIAPDRASFFRGESLGVQSDVPLDSGCEGDCFHSGGTIVSS
A/chicken/Jalisco/CPA1/2012                           ...............................................T...........................................................................I..................N.......
A/Mexico/InDRE7218/2012                               ...............................................T...........................................................................I..................N.......
A/chicken/Jalisco/12283/2012                          ..............................................DT...........................................................................I..................N.......
A/cinnamon-teal/Mexico/2817/2016                      ...............................................D......................................................................................................
A/wild_bird/Chiapas/CPA-12288-15-CENASA-96131/2015NP................T.....R......................T.........D..........L......................................................I.I................N.......
A/wild_bird/Chiapas/CPA-12858-15-CENASA-96131/2015NP................T.....R......................T.........D..........L......................................................I.I................N.......
A/wild_bird/Chiapas/CPA-13000-15-CENASA-96131/2015NP................T.....R......................T.........D..........L......................................................I.I................N.......
/Ortalis_vetula/Chiapas/CPA-13808-15-CENASA-96131/NP................T.....R......................T.........D..........L......................................................I.I................N.......
A/Amazona_albifrons/Chiapas/CPA-13813-15-CENASA-96NP................T.....R......................T.........D..........L......................................................I.I................N.......
A/Ortalis_vetula/Chiapas/CPA-14309-15-CENASA-96131NP................T.....R......................T.........D..........L......................................................I.I................N.......
A/Ortalis_vetula/Chiapas/CPA-14326-15-CENASA-96131NP................T.....R......................T.........D..........L......................................................I.I................N.......
A/Ortalis_vetula/Chiapas/CPA-14177-15-CENASA-96131NP................T.....R......................T.........D..........L......................................................I.I................N.......
A/Ortalis_vetula/Chiapas/CPA-14469-15-CENASA-96131NP................T.....R......................T.........D..........L......................................................I.I................N.......
A/Ortalis_vetula/Chiapas/CPA-14539-15-CENASA-96131NP................T.....R......................T.........D..........L......................................................I.I................N.......
A/Ortalis_vetula/Chiapas/CPA-14774-15-CENASA-96131NP................T.....R......................T.........D..........L......................................................I.I................N.......
A/Ortalis_vetula/Chiapas/CPA-15350-15-CENASA-96131NP................T.....R......................T.........D..........L......................................................I.I................N.......
A/Ortalis_vetula/Chiapas/CPA-15172-15-CENASA-96131NP................T.....R......................T.........D..........L......................................................I.I................N.......
A/Ortalis_vetula/Chiapas/CPA-16029-15-CENASA-96131NP................T.....R......................T.........D..........L......................................................I.I................N.......
A/wild_bird/Chiapas/CPA-13973-15-CENASA-96131/2015NP................T.....R......................T.........D..........L......................................................I.I................N.......
A/Ortalis_vetula/Chiapas/CPA-14466-15-CENASA-96131NP................T.....R......................T.........D..........L......................................................I.I................N.......
A/Ortalis_vetula/Chiapas/CPA-14472-15-CENASA-96131NP................T.....R......................T.........D..........L......................................................I.I................N.......
A/Ortalis_vetula/Chiapas/CPA-15868-15-CENASA-96131NP................T.....R......................T.........D..........L......................................................I.I................N.......
A/Ortalis_vetula/Chiapas/CPA-15874-15-CENASA-96131NP................T.....R......................T.........D..........L......................................................I.I................N.......
A/Ortalis_vetula/Chiapas/CPA-16988-15-CENASA-96131NP................T.....R......................T.........D..........L......................................................I.I................N.......
A/wild_bird/Chiapas/CPA-15205-15-CENASA-96131/2015NP................T.....R......................T.........D..........L.....N................................................I.I................N.......
A/chicken/Puebla/CPA_07421/2015                       NP................T.....R......D...............T.........D..........M......................................................I.I................N.......
A/chicken/Puebla/CPA-28973/2015                       NP................T.....R......................T.........D..........M.....I............................................K...I.I................N.......
A/chicken/Jalisco/CPA-01655-16-CENASA-95076/2016      SP................T..K..Q......................T.........D..........L...........K..........................................I.I........D.......N.......
A/chicken/Puebla/CPA-03191-16-CENASA-95076/2016       SP................T..K..Q......................T.........D..........L...........K..........................................I.I........D.......N.......
A/chicken/Jalisco/CPA-01858-16-CENASA-95294/2016      SP................T..K..Q......................T.........D..........L...........K..........................................I.I........D.......N.......
A/chicken/Jalisco/CPA-01859-16-CENASA-95294/2016      SP................T..K..Q......................T.........D..........L...........K..........................................I.I........D.......N.......
A/chicken/Jalisco/CPA-01863-16-CENASA-95294/2016      SP................T..K..Q......................T.........D..........L...........K..........................................I.I........D.......N.......
A/chicken/Jalisco/CPA-01864-16-CENASA-95294/2016      SP................T..K..Q......................T.........D..........L...........K..........................................I.I........D.......N.......
A/chicken/Jalisco/CPA-01861-16-CENASA-95294/2016      SP................T..K..Q......................T.........D..........L...........K..........................................I.I........D.......N.......
A/chicken/Jalisco/PAVX17170/2017                      SP...T.......S.E..T..N..R......................T.........D..........L......L....K..........................................I.I........D.......N.......
A/chicken/Guanajuato/CPA-06664-18-VS/2018             S....T............T..N..R......................T.........D..........L.....I..P..K..........................................I.I........D.......N......A
A/chicken/Jalisco/05/2018                             S....T............T..N..R......................T.........D..........L.....I..P..K................................G.........I.I........D.......N......A
A/chicken/Mexico_State/09-06/2009                     S....T............T..N..R......................T.........D..........L.....I..S..K..........................................I.I........N.......N.......
A/chicken/Mexico/Jalisco_CPA-06078-19/2019            SP...............VT..N..R......................T.........D..........L.....I..P.............................................I.I........D.......N.......
A/chicken/Jalisco/16-07/2023                          SP................T..N..L.....K................T.........D..........L.....I..P.............................................I.I............G...N.......
A/chicken/Puebla/02/2019                              S.................T..N..R....T.................T....................L.....I..P..K..........................................I.I........D.......N.......
A/chicken/Queretaro/01/2018                           S.................T..N..R....T.................T....................L.....I..P..K..........................................I.I........D.......N.......
A/chicken/San_Luis_Potosi/CPA-00562-22/2022           .P...............ET..N..R............V.........T.......R.D..........L.....I..P...................................V.....K...I.I.......FD.......N.......
A/chicken/Jalisco/10/2021                             .P...............ET..N..Q............V.........T.......R.D..........L.....I..P.........................................K...I.I.......FD.......N.......
A/chicken/Aguascalientes/10/2021                      .P...............ET..N..Q............V.........T.......R.D..........L.....I..P.........................................K...I.I.......FD.......N.......
A/chicken/Aguascalientes/12/2021                      NP...............ET..NI.Q............V.........T.........D..........L.....I..P...................................V.....K...I.........FD.......N.......
A/chicken/Mexico_State/21-14/2014                     NP...............ET..N..Q.....K......V.........T.........D..........L.....I..P.........................................K...I.I.......FD.......N.......
A/chicken/Puebla/CPA-02247-22/2022                    S.................T..N..R....L................VD.........D..........L.....I..P..K................................V.......K.I.I........N.......N.......
A/chicken/Puebla/CPA-02527-22/2022                    S.................T..N..R....L................VD.........D..........L.....I..P..K................................V.......K.I.I........N.......N.......
A/chicken/Coahuila/CPA-03045-22/2022                  K....T.......T...VT..N..L..T...S.....V.........T.........D..........L.....I..P..K......................................K...I.I.......FD.......N.......
A/chicken/Durango/CPA-03739-22/2022                   K....T.......T...VT..N..L..T...S.....V.........T.........D..........L.....I..P..K......................................K...I.I.......FD.......N.......
A/chicken/Durango/CPA-03922-22/2022                   K....T.......T...VT..N..L..T...S.....V.........T.........D..........L.....I..P..K......................................K...I.I.......FD.......N.......
A/chicken/Coahuila/CPA-03166-22/2022                  K....T.......T...VT..N..L..T...S.....V.........T.........D..........L.....I..P..K......................................K...I.I.......FD.......N....M..
A/chicken/Coahuila/CPA-03703-22/2022                  K....T.......T...VT..N..L..T...S.....V.........T.........D..........L.....I..P..K......................................K...I.I.......FD.......N.......
A/chicken/Coahuila/CPA-03046-22/2022                  K....T.......T...VT..N..L..T.........V.........T.........D..........L.....I..P..K......................................K...I.I.......FD.......N.......
A/chicken/Durango/CPA-03482-22/2022                   K....T.......T...VT..N..L..T.........V.........T.........D..........L.....I..P..K....................I.................K...I.I.......FD.......N.......
A/chicken/Durango/CPA-03865-22/2022                   K....T.......T...VT..N..L..T.........V.........T.........D..........L.....I..P..K....................I.................K...I.I.......FD.......N.......
A/chicken/Durango/CPA-03872-22/2022                   K....T.......T...VT..N..L..T.........V.........T.........D..........L.....I..P..K....................I.................K...I.I.......FD.......N.......
A/chicken/Guanajuato/CPA-01914-22/2022                K....T.......T...VT..N..L..T.........V.........T.........D..........L.....I..P..K......................................K...I.I.......FD.......N.......
A/chicken/Aguascalientes/4-10/2023                    K....T.......T...VT..N..L..T.........V.........T.........D..........L.....I..P..K......................................K...I.I.......FD.......N.......
A/chicken/Jalisco/11/2023                             K....T.......T...VT..N..L..T.........V.........T....................L.....I..P..K......................................K...I.I.......FD.......N.......
A/chicken/Durango/CPA-03276-22/2022                   K....T.......T...VT..N..L..T...S.....V.........T.........D..........L.....I..P..K................S.....................K...V.I.......FD.......N.......
A/chicken/Jalisco/CPA-01178-22/2022                   K....T.......T...VT..N..L..T.........V.........T.........D..........L.....I..P..K......................................K...I.I.......FD.......N.......
A/chicken/Guanajuato/CPA-01819-22/2022                K....T.......T...VT..N.NL..T..K................T.........D..........L.....I..P..K......................................K...I.I.......F........N.......
A/chicken/Jalisco/CPA-02681-22/2022                   K....T.......T...VT..N.NL..T..K................T.........D..........L...I.I..P..K......................................K...I.I.......F........N.......
A/chicken/Jalisco/86-1-03/2022                        K....T.......T...VT..N.NL..T..K................T.........D..........L.....I..P..K......................................K...I.I.......F........N.......
A/chicken/Guanajuato/CPA-03095-22/2022                K....T.......T...VT..N.NL..T..K................T.........D..........L.....I..P..K......................................K...I.I.......F........N.......
A/chicken/Guanajuato/07437-15/2015                    NP...........D....T.....M........S.............T.........D.F........L........P..K..........................................I.I.......P..K.....N.......
A/chicken/Guanajuato/CPA-02921-16-CENASA-_95294/20NP................T.....M.......RS.............I.........D.F........L........P..K..........................................I.I.......P..K.....N.......
A/chicken/Jalisco/CPA-04173-16-CENASA-95294/2016      NP................T.....M.......RS.............I.........D.F........L........P..K..........................................I.I.......P..K.....N.......
A/chicken/Jalisco/7LG/2017                            NP...............ET.....M......K.S.............T.........D.F........L...........K..........................................I.I.......P..K.....N.......
A/chicken/Jalisco/7DIEGO/2017                         NP................T.....M........S.............I.........D.F........L...........K..........................................V.I.......P..K.....N.......
A/chicken/Mexico/CPA-37905/2015                       NP................T.....M........S.............T.........G.F........L........P..K..........................................I.I.......P..K.N...N.......
A/chicken/Puebla/CPA-03309-16-CENASA-95076/2016       NP................T.....M........S............TT.........D.F........L...........K..........................................I.I...A...PD.K.....N.......
A/chicken/Puebla/CPA-04451-16-CENASA-95294/2016       NP................T.....M........S............TT.........D.F........L...........K..........................................I.I...A...PD.K.....N.......
A/chicken/Puebla/CPA-04760-16-CENASA-95294/2016       NP................T.....M........S............TT.........D.F........L...........K..........................................I.I...A...PD.K.....N.......
A/chicken/Puebla/CPA-02457-16-CENASA-95294/2016       NP................T.....M........S............TT.........D.F........L...........K..........................................I.I...A...PD.K.....N.......
A/chicken/Puebla/CPA-03191/2016                       NP................T.....M........S............TT.........D.F........L...........K..........................................I.I...A...PD.K.....N.......
A/chicken/Puebla/CPA-04148-16-CENASA-95294/2016       NP................T.....M........S............TT.........D.F........L...........K..........................................I.I...A...PD.K.....N.......
A/chicken/Guanajuato/CPA-07669-16-VS/2016             NP......R.........T.....M........S............TT.........D.F........L...........K..........................................I.I...A...PD.K.....N.......
A/chicken/Jalisco/716/2017                            NP...V...........ET..............S.............T...........F........L...N.......K................................K.........I.I.......P..K.....N.......
```

```
                                                                                         →       ←
A/cinnamon_teal/Mexico/2817/2006 LPFQNINPRAVGKCPRYVKQTSLLLATGMRNVPENPK--------TRGLFGAIAGFIENGWEGLIDGWYGFRHQNAQGEGTAADYKSTQSAIDQITGKLNRLIDKTNQQFELIDNEFSEIEQQIGNVINWTRDSMTEVWSYNAELLVAME
A/chicken/Jalisco/CPA1/2012 .........T...........................DRKSRHRR.........................................................................................................
A/Mexico/InDRE7218/2012 .........T...........................DRKSRHRR.........................................................................................................
A/chicken/Jalisco/12283/2012 .........T...........................DRKSRHRR.........................................................................................................
A/cinnamon-teal/Mexico/2817/2016 .........T...........................--------..................................................................M.....N................................
A/wild_bird/Chiapas/CPA-12288-15-CENASA-96131/2015.........T...........................DRKSRHRR............................................Y........................................................I...
A/wild_bird/Chiapas/CPA-12858-15-CENASA-96131/2015.........T...........................DRKSRHRR............................................Y........................................................I...
A/wild_bird/Chiapas/CPA-13000-15-CENASA-96131/2015.........T...........................DRKSRHRR............................................Y........................................................I...
/Ortalis_vetula/Chiapas/CPA-13808-15-CENASA-96131/.........T...........................DRKSRHRR............................................Y........................................................I...
A/Amazona_albifrons/Chiapas/CPA-13813-15-CENASA-96.........T...........................DRKSRHRR............................................Y........................................................I...
A/Ortalis_vetula/Chiapas/CPA-14309-15-CENASA-96131.........T...........................DRKSRHRR............................................Y........................................................I...
A/Ortalis_vetula/Chiapas/CPA-14326-15-CENASA-96131.........T...........................DRKSRHRR............................................Y........................................................I...
A/Ortalis_vetula/Chiapas/CPA-14177-15-CENASA-96131.........T...........................DRKSRHRR............................................Y........................................................I...
A/Ortalis_vetula/Chiapas/CPA-14469-15-CENASA-96131.........T...........................DRKSRHRR............................................Y........................................................I...
A/Ortalis_vetula/Chiapas/CPA-14539-15-CENASA-96131.........T...........................DRKSRHRR............................................Y........................................................I...
A/Ortalis_vetula/Chiapas/CPA-14774-15-CENASA-96131.........T...........................DRKSRHRR............................................Y........................................................I...
A/Ortalis_vetula/Chiapas/CPA-15350-15-CENASA-96131.........T...........................DRKSRHRR............................................Y........................................................I...
A/Ortalis_vetula/Chiapas/CPA-15172-15-CENASA-96131.........T...........................DRKSRHRR............................................Y........................................................I...
A/Ortalis_vetula/Chiapas/CPA-16029-15-CENASA-96131.........T...........................DRKSRHRR............................................Y........................................................I...
A/wild_bird/Chiapas/CPA-13973-15-CENASA-96131/2015.........T...........................DRKSRHRR............................................Y........................................................I...
A/Ortalis_vetula/Chiapas/CPA-14466-15-CENASA-96131.........T...........................DRKSRHRR............................................Y........................................................I...
A/Ortalis_vetula/Chiapas/CPA-14472-15-CENASA-96131.........T...........................DRKSRHRR............................................Y........................................................I...
A/Ortalis_vetula/Chiapas/CPA-15868-15-CENASA-96131.........T...........................DRKSRHRR............................................Y........................................................I...
A/Ortalis_vetula/Chiapas/CPA-15874-15-CENASA-96131.........T...........................DRKSRHRR............................................Y........................................................I...
A/Ortalis_vetula/Chiapas/CPA-16988-15-CENASA-96131.........T...........................DRKSRHRR............................................Y........................................................I...
A/wild_bird/Chiapas/CPA-15205-15-CENASA-96131/2015.........T...........................DKKSRHRR............................................Y........................................................I...
A/chicken/Puebla/CPA_07421/2015 .........T.........................S.DMKSRHRK............................................Y........................................................I...
A/chicken/Puebla/CPA-28973/2015 .........T.........................S.DMKSRHRK............................................Y........................................................I...
A/chicken/Jalisco/CPA-01655-16-CENASA-95076/2016 .........T...........................GKKSRHRK...............................................N.....................................................IS..
A/chicken/Puebla/CPA-03191-16-CENASA-95076/2016 .........T...........................GKKSRHRK...............................................N.....................................................IS..
A/chicken/Jalisco/CPA-01858-16-CENASA-95294/2016 .........T...........................GKKSRHRK...............................................N.....................................................IS..
A/chicken/Jalisco/CPA-01859-16-CENASA-95294/2016 .........T...........................GKKSRHRK...............................................N.....................................................IS..
A/chicken/Jalisco/CPA-01863-16-CENASA-95294/2016 .........T...........................GKKSRHRK...............................................N.....................................................IS..
A/chicken/Jalisco/CPA-01864-16-CENASA-95294/2016 .........T...........................GKKSRHRK...............................................N.....................................................IS..
A/chicken/Jalisco/CPA-01861-16-CENASA-95294/2016 .........T...........................GKKSRHRK...............................................N.....................................................IS..
A/chicken/Jalisco/PAVX17170/2017 .........T...........................GRKSRHRK...............................................N.....................................................IS..
A/chicken/Guanajuato/CPA-06664-18-VS/2018 .........T...........................GRKSRHRK...............................................N.....................................................IS..
A/chicken/Jalisco/05/2018 .........T...........................GRKSRHRK...............................................N.....................................................IS..
A/chicken/Mexico_State/09-06/2009 .........T...........................GRKSRHRK...............................................N.....................................................IS..
A/chicken/Mexico/Jalisco_CPA-06078-19/2019 .........T...........................GRKSRHRK...............................................N.....................................................IS..
A/chicken/Jalisco/16-07/2023 .........T...........................GRESRHRK...............................................N.....................................................IS..
A/chicken/Puebla/02/2019 .........T...........................GRKSRHRK...............................................N.....................................................IS..
A/chicken/Queretaro/01/2018 .........T...........................GRKSRHRK...............................................N.....................................................IS..
A/chicken/San_Luis_Potosi/CPA-00562-22/2022 .........T..........K................GRKSLHRK...............................................N.........................................V............S..
A/chicken/Jalisco/10/2021 .........T..........K................GRKSLHRK...............................................N......................................................S..
A/chicken/Aguascalientes/10/2021 .........T..........K................GRKSLHRK...............................................N......................................................S..
A/chicken/Aguascalientes/12/2021 .........T..........A................GRKSLHRK...............................................N.....................................................IS..
A/chicken/Mexico_State/21-14/2014 .........T...........................GRESRHRK...............................................N.....................................................IS..
A/chicken/Puebla/CPA-02247-22/2022 .........TI..........................GGKSRHRK...............................................N..................M..................................IS..
A/chicken/Puebla/CPA-02527-22/2022 .........TI..........................GGKSRHRK...............................................N..................M..................................IS..
A/chicken/Coahuila/CPA-03045-22/2022 .........T..........K................SRKSQHRK...............................................N............................................L........IS..
A/chicken/Durango/CPA-03739-22/2022 .........T..........K................SRKSQHRK...............................................N............................................L........IS..
A/chicken/Durango/CPA-03922-22/2022 .........T..........K................SRKSQHRK...............................................N............................................L........IS..
A/chicken/Coahuila/CPA-03166-22/2022 .........T..........K................SRKSQHRK...............................................N............................................L........IS..
A/chicken/Coahuila/CPA-03703-22/2022 .........T..........K................SRKSQHRK...............................................N............................................L........IS..
A/chicken/Coahuila/CPA-03046-22/2022 .........T..........K................SRKSQHRK...............................................N............................................L........IS..
A/chicken/Durango/CPA-03482-22/2022 .........T..........K................SRKSQHRK...............................................N............................................L........IS..
A/chicken/Durango/CPA-03865-22/2022 .........T..........K................SRKSQHRK...............................................N............................................L........IS..
A/chicken/Durango/CPA-03872-22/2022 .........T..........K................SRKSQHRK...............................................N............................................L........IS..
A/chicken/Guanajuato/CPA-01914-22/2022 .........T..........K................SRKSQHRK...............................................N............................................L........IS..
A/chicken/Aguascalientes/4-10/2023 .........T..........K................SRKSQHRK...............................................N............................................L........IS..
A/chicken/Jalisco/11/2023 .........T..........K................SRKSQHRK...............................................N............................................L........IS..
A/chicken/Durango/CPA-03276-22/2022 .........T..........K................SRKSQHRK...............................................N............................................L........IS..
A/chicken/Jalisco/CPA-01178-22/2022 .........T..........K................SRKSQHRK...............................................N............................................L........IS..
A/chicken/Guanajuato/CPA-01819-22/2022 .........T..........K................SRKSQHRK...............................................N.....................................................IS..
A/chicken/Jalisco/CPA-02681-22/2022 .........T..........K................SRKSRHRK...............................................N.....................................................IS..
A/chicken/Jalisco/86-1-03/2022 .........T..........K................SRKSRHRK...............................................N.....................................................IS..
A/chicken/Guanajuato/CPA-03095-22/2022 .........T..........K................SRKSRHRK...............................................N........................N............................IS..
A/chicken/Guanajuato/07437-15/2015 .........T..........P................DRKSRHRR...............................................N.....................................................I...
A/chicken/Guanajuato/CPA-02921-16-CENASA-_95294/20.........T..........P................DRKSRHRR...............................................N.....................................................I...
A/chicken/Jalisco/CPA-04173-16-CENASA-95294/2016 .........T..........P................DRKSRHRR...............................................N.....................................................I...
A/chicken/Jalisco/7LG/2017 .........T..........P................DRKSRHRR...............................................N............................................I........I...
A/chicken/Jalisco/7DIEGO/2017 .........T..........P................DRKGRHRR...............................................N.....................................................I...
A/chicken/Mexico/CPA-37905/2015 .........T..........P................DRKRRHRR...............................................N.....................................................I...
A/chicken/Puebla/CPA-03309-16-CENASA-95076/2016 .........T...........................DRKNRHRR...............................................N.....................................................I...
A/chicken/Puebla/CPA-04451-16-CENASA-95294/2016 .........T...........................DRKNRHRR...............................................N.....................................................I...
A/chicken/Puebla/CPA-04760-16-CENASA-95294/2016 .........T...........................DRKNRHRR...............................................N.....................................................I...
A/chicken/Puebla/CPA-02457-16-CENASA-95294/2016 .........T...........................DRKNRHRR...............................................N.....................................................I...
A/chicken/Puebla/CPA-03191/2016 .........T...........................DRKNRHRR...............................................N.....................................................I...
A/chicken/Puebla/CPA-04148-16-CENASA-95294/2016 .........T...........................DRKNRHRR...............................................N.....................................................I...
A/chicken/Guanajuato/CPA-07669-16-VS/2016 .........T...........................DRKNRHRR...............................................N.....................................................I...
A/chicken/Jalisco/716/2017 .........T..........P................DWKSRHRR...............................................N........................R............................I...
```

```
A/cinnamon_teal/Mexico/2817/2006                      NQHTIDLADSEMNKLYERVRKQLRENAEEDGTGCFEIFHKCDDQCMESIRNNTYDHAQYRTESLQNRIQIDPVKLSSGYKDIILWFSFGASCFLLLAIAMGLVFICIKNGNMRCTICI
A/chicken/Jalisco/CPA1/2012                           ........................................................T...A.........................................................
A/Mexico/InDRE7218/2012                               ........................................................T...A.........................................................
A/chicken/Jalisco/12283/2012                          ........................................................T...A.........................................................
A/cinnamon-teal/Mexico/2817/2016                      ........................................................T......................................I......................
A/wild_bird/Chiapas/CPA-12288-15-CENASA-96131/2015    .......T................................................N...A.........................................................
A/wild_bird/Chiapas/CPA-12858-15-CENASA-96131/2015    .......T................................................N...A.........................................................
A/wild_bird/Chiapas/CPA-13000-15-CENASA-96131/2015    .......T................................................N...A.........................................................
A/Ortalis_vetula/Chiapas/CPA-13808-15-CENASA-96131    .......T................................................N...A.........................................................
A/Amazona_albifrons/Chiapas/CPA-13813-15-CENASA-96    .......T................................................N...A.........................................................
A/Ortalis_vetula/Chiapas/CPA-14309-15-CENASA-96131    .......T................................................N...A.........................................................
A/Ortalis_vetula/Chiapas/CPA-14326-15-CENASA-96131    .......T................................................N...A.........................................................
A/Ortalis_vetula/Chiapas/CPA-14177-15-CENASA-96131    .......T................................................N...A.........................................................
A/Ortalis_vetula/Chiapas/CPA-14469-15-CENASA-96131    .......T................................................N...A.........................................................
A/Ortalis_vetula/Chiapas/CPA-14539-15-CENASA-96131    .......T................................................N...A.........................................................
A/Ortalis_vetula/Chiapas/CPA-14774-15-CENASA-96131    .......T................................................N...A.........................................................
A/Ortalis_vetula/Chiapas/CPA-15350-15-CENASA-96131    .......T................................................N...A.........................................................
A/Ortalis_vetula/Chiapas/CPA-15172-15-CENASA-96131    .......T................................................N...A.........................................................
A/Ortalis_vetula/Chiapas/CPA-16029-15-CENASA-96131    .......T................................................N...A.........................................................
A/wild_bird/Chiapas/CPA-13973-15-CENASA-96131/2015    .......T................................................N...V.........................................................
A/Ortalis_vetula/Chiapas/CPA-14466-15-CENASA-96131    .......T................................................N...V.........................................................
A/Ortalis_vetula/Chiapas/CPA-14472-15-CENASA-96131    .......T................................................N...V.........................................................
A/Ortalis_vetula/Chiapas/CPA-15868-15-CENASA-96131    .......T................................................N...A...................N.....................................
A/Ortalis_vetula/Chiapas/CPA-15874-15-CENASA-96131    .......T................................................N...A...................N.....................................
A/Ortalis_vetula/Chiapas/CPA-16988-15-CENASA-96131    .......T................................................N...A...................N.....................................
A/wild_bird/Chiapas/CPA-15205-15-CENASA-96131/2015    .......T................................................N...A.........................................................
A/chicken/Puebla/CPA_07421/2015                       .......T................................................T...A.........................................................
A/chicken/Puebla/CPA-28973/2015                       .......T................................................T...A.........................................................
A/chicken/Jalisco/CPA-01655-16-CENASA-95076/2016      ........................................................T...A.........E...............................................
A/chicken/Puebla/CPA-03191-16-CENASA-95076/2016       ........................................................T...A.........E...............................................
A/chicken/Jalisco/CPA-01858-16-CENASA-95294/2016      ........................................................T...A.........E...............................................
A/chicken/Jalisco/CPA-01859-16-CENASA-95294/2016      ........................................................T...A.........E...............................................
A/chicken/Jalisco/CPA-01863-16-CENASA-95294/2016      ........................................................T...A.........E...............................................
A/chicken/Jalisco/CPA-01864-16-CENASA-95294/2016      ........................................................T...A.........E...............................................
A/chicken/Jalisco/CPA-01861-16-CENASA-95294/2016      ........................................................T...A.........E...............................................
A/chicken/Jalisco/PAVX17170/2017                      ........................................................T...A.........E...............................................
A/chicken/Guanajuato/CPA-06664-18-VS/2018             ........................................................T...A.........E...............................................
A/chicken/Jalisco/05/2018                             ........................................................T...A.........E...............................................
A/chicken/Mexico_State/09-06/2009                     .........................................N..............T...A.........E...............................................
A/chicken/Mexico/Jalisco_CPA-06078-19/2019            ........................................................T...A.........E...............................................
A/chicken/Jalisco/16-07/2023                          ........................................................T...A.........E...............................................
A/chicken/Puebla/02/2019                              ........................................................T...A.........E...............................................
A/chicken/Queretaro/01/2018                           ........................................................T...A.........E...............................................
A/chicken/San_Luis_Potosi/CPA-00562-22/2022           ........................................................T...A.........E...............................................
A/chicken/Jalisco/10/2021                             ........................................................T...A.........E...............................................
A/chicken/Aguascalientes/10/2021                      ........................................................T...A.........E...............................................
A/chicken/Aguascalientes/12/2021                      ...........................................H............T...A.........E...............................................
A/chicken/Mexico_State/21-14/2014                     ........................................................T...A.........E...............................................
A/chicken/Puebla/CPA-02247-22/2022                    ........................................................T...A.........E......................I........................
A/chicken/Puebla/CPA-02527-22/2022                    ........................................................T...A.........E......................I........................
A/chicken/Coahuila/CPA-03045-22/2022                  ........................................................T...A.........E...............................................
A/chicken/Durango/CPA-03739-22/2022                   ........................................................T...A.........E...............................................
A/chicken/Durango/CPA-03922-22/2022                   ........................................................T...A.........E...............................................
A/chicken/Coahuila/CPA-03166-22/2022                  ........................................................T...A.........E...............................................
A/chicken/Coahuila/CPA-03703-22/2022                  ........................................................T...A.........E...............................................
A/chicken/Coahuila/CPA-03046-22/2022                  ........................................................T...A.........E...............................................
A/chicken/Durango/CPA-03482-22/2022                   ........................................................T...A.........E...............................................
A/chicken/Durango/CPA-03865-22/2022                   ........................................................T...A.........E...............................................
A/chicken/Durango/CPA-03872-22/2022                   ..........................................N.............T...A.........E...............................................
A/chicken/Guanajuato/CPA-01914-22/2022                ........................................................T...A.........E...............................................
A/chicken/Aguascalientes/4-10/2023                    ........................................................T...A.........E...............................................
A/chicken/Jalisco/11/2023                             ........................................................T...A.........E...............................................
A/chicken/Durango/CPA-03276-22/2022                   ........................................................T...A.........E...............................................
A/chicken/Jalisco/CPA-01178-22/2022                   ........................................................T...A.........E...............................................
A/chicken/Guanajuato/CPA-01819-22/2022                ........................................................T...A.........E...............................................
A/chicken/Jalisco/CPA-02681-22/2022                   ........................................................T...A.........E...............................................
A/chicken/Jalisco/86-1-03/2022                        ........................................................T...A.........E...............................................
A/chicken/Guanajuato/CPA-03095-22/2022                ........................................................T...A.........E...V...........................................
A/chicken/Guanajuato/07437-15/2015                    ....................................M...................T...A.........................................................
A/chicken/Guanajuato/CPA-02921-16-CENASA-_95294/20    ....................................M...................T...A.........................................................
A/chicken/Jalisco/CPA-04173-16-CENASA-95294/2016      ....................................M...................T...A.........................................................
A/chicken/Jalisco/7LG/2017                            ........................................................T...A.........................................................
A/chicken/Jalisco/7DIEGO/2017                         ....................................M...................T...A.........................................................
A/chicken/Mexico/CPA-37905/2015                       ....................R...............M...................T...A.........................................................
A/chicken/Puebla/CPA-03309-16-CENASA-95076/2016       ........................................................T...A.........................................................
A/chicken/Puebla/CPA-04451-16-CENASA-95294/2016       ........................................................T...A.........................................................
A/chicken/Puebla/CPA-04760-16-CENASA-95294/2016       ........................................................T...A.........................................................
A/chicken/Puebla/CPA-02457-16-CENASA-95294/2016       ........................................................T...A.........................................................
A/chicken/Puebla/CPA-03191/2016                       ........................................................T...A.........................................................
A/chicken/Puebla/CPA-04148-16-CENASA-95294/2016       ........................................................T...A.....................V...................................
A/chicken/Guanajuato/CPA-07669-16-VS/2016             ........................................................T...A.........................................................
A/chicken/Jalisco/716/2017                            ....................................M...................T...A................................V........................
```
