# Supplementary material for: Genetic and Serological Analysis of H7N3 Avian Influenza Viruses in Mexico for Pandemic Risk Assessment
Source: Viruses. 2025 Oct 15;17(10):1376. doi: 10.3390/v17101376 (PMC12567671; doi:10.3390/v17101376)
Supplement: Supplementary file 1 [file viruses-17-01376-s001.zip › viruses-3852067 Supplementary Materials.pdf]

Figure S1: Alignment of hemagglutinin amino acid sequences of Mexican H7N3 viruses. The amino acid position is based on H7 HA numbering where methionine (M) encoded by ATG is at amino acid position number 1. Differences in amino acids are relative to the A/cinnamon_teal/Mexico/2817/2006 (H7N3) strain. The receptor binding site comprising the 130 loop, 190 helix and 220 loop are represented bold underline. The glycosylation sites are highlighted in light blue. The red letters indicate conserved amino acids in the RBS. The cleavage site between HA1–HA2 is indicated by bold arrows.

Figure S2: Three-dimensional modeling of avian influenza A(H7N3) hemagglutinin from Mexican isolates. Structures of strains A/Chicken/Jalisco/CPA1/2012, A/Chicken/Guanajuato/CPA-07669-16-VS/2016, and A/Chicken/Mexico/Jalisco_CPA-06078-19/2019 are shown (top and side views). The 2012 strain is presented as reference, showing no glycosylation sites at the indicated positions. The 2016 strain exhibits substitutions A143T (pink), A169T (purple), and P184S (violet), generating NGT, NAT, and NKS motifs. The 2019 strain displays A169T (purple) and Q172N (pink), giving rise to NAT and NMT motifs. Models were generated with SwissModel and visualized in PyMOL.

Figure S3:Frequency of amino acids across the hemagglutinin HA1 domain. Green star indicates amino acid position 91; black stars indicates amino acid positions 140 and 166.
Figure S4: Phylogenetic trees of all segments.

Table S1. Geographic and age distribution of serum samples analyzed by hemagglutination inhibition and microneutralization assays, Mexico, 2017-2019 and 2022-2025.

State	2017	2018	2019	2022	2023	2024	2025
**Aguascalientes**					51	5	
**Baja California**				3			
**Chihuahua**						4	21
**Chiapas**				8			
**Coahuila**				6			34
**Distrito Federal**		1	4	1		29	6
**Durango**							83
**Guanajuato**		9	13		40	12	
**Hidalgo**			1				
**Jalisco**	41				5	7	13
**México**			5	8		36	
**Michoacán**						2	
**Morelia**			1				
**Nuevo León**				61			147
**Puebla**			1		4	66	
**Qerétaro**		11	13				
**San Luis Potosí**						8	
**Sonora**				135	65	20	15
**Tabasco**			1				
**Tamaulipas**				6			
**Veracruz**					30	12	
**Yucatan**				15	42		
**Zacatecas**			1			1	
**Age Group, years**							
**0-9**	1			14	1	7	20
**10-19**	1		4	14	1	13	19
**20-29**	10		5	62	62	52	59
**30-39**	11	11	7	53	53	58	85
**40-49**	16	4	13	59	59	36	64
**50-59**	2	6	8	36	49	22	52
**60-69**			2	3	11	10	15
**70+**			1	2	1	4	5
**Total**	**41**	**21**	**40**	**243**	**237**	**202**	**319**
