# Supplementary figures and images for: Genetic and Serological Analysis of H7N3 Avian Influenza Viruses in Mexico for Pandemic Risk Assessment
Source: Viruses. 2025 Oct 15;17(10):1376. doi: 10.3390/v17101376 (PMC12567671; doi:10.3390/v17101376)

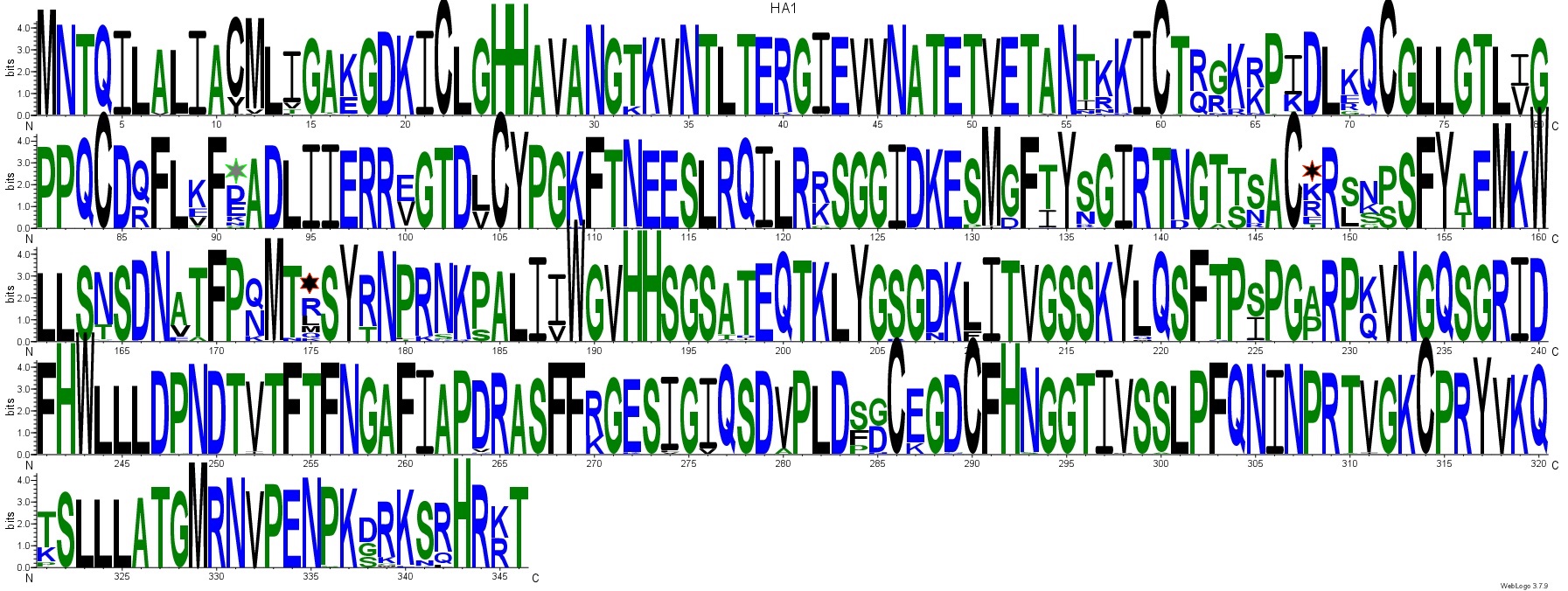

Supplement: Supplementary file 1 [file viruses-17-01376-s001.zip › Figure S3. HA1 weblogo.jpeg]
